# Markers of Microbial Translocation and Immune Activation Predict Cognitive Processing Speed in Heavy-Drinking Men Living with HIV

**DOI:** 10.3390/microorganisms5040064

**Published:** 2017-09-21

**Authors:** Mollie A. Monnig, Christopher W. Kahler, Patricia A. Cioe, Peter M. Monti, Kenneth H. Mayer, David W. Pantalone, Ronald A. Cohen, Bharat Ramratnam

**Affiliations:** 1 Center for Alcohol and Addiction Studies, Brown University School of Public Health, Providence, RI 02912, USA; christopher_kahler@brown.edu (C.W.K.); patricia_cioe@brown.edu (P.A.C.); peter_monti@brown.edu (P.M.M.); 2 The Fenway Institute, Fenway Health, Boston, MA 02215, USA; kmayer@fenwayhealth.org (K.H.M.); david.pantalone@umb.edu (D.W.P.); 3 Department of Medicine, Beth Israel Deaconness Medical Center/Harvard Medical School, Boston, MA 02215, USA; 4 Department of Psychology, University of Massachusetts, Boston, MA 02125, USA; 5 Department of Clinical and Health Psychology, University of Florida, Gainesville, FL 32603, USA; roncohen@ufl.edu; 6 COBRE Center for Cancer Research, Rhode Island and Miriam Hospitals, Lifespan Health System, Providence, RI 02903, USA; bramratnam@lifespan.org; 7 Division of Infectious Diseases, Department of Medicine, Warren Alpert Medical School, Brown University, Providence, RI 02903, USA

**Keywords:** gut-brain axis, HIV infection, alcohol use disorder, heavy drinking, inflammation, microbial translocation, monocyte activation, cognition, processing speed

## Abstract

HIV infection and alcohol use disorder are associated with deficits in neurocognitive function. Emerging evidence points to pro-inflammatory perturbations of the gut-brain axis as potentially contributing to neurocognitive impairment in the context of HIV and chronic heavy alcohol use. This study examined whether plasma markers of microbial translocation (LPS) from the gastrointestinal tract and related immune activation (sCD14, EndoCAb) were associated with neurocognition in 21 men living with HIV who were virally suppressed on antiretroviral therapy. All participants met federal criteria for heavy drinking and were enrolled in a randomized controlled trial (RCT) of a brief alcohol intervention. This secondary analysis utilized blood samples and cognitive scores (learning, memory, executive function, verbal fluency, and processing speed) obtained at baseline and three-month follow-up of the RCT. In generalized estimating equation models, LPS, sCD14, and EndoCAb individually were significant predictors of processing speed. In a model with all biomarkers, higher LPS and sCD14 both remained significant predictors of lower processing speed. These preliminary findings suggest that inflammation stemming from HIV and/or alcohol could have negative effects on the gut-brain axis, manifested as diminished processing speed. Associations of microbial translocation and immune activation with processing speed in heavy-drinking PLWH warrant further investigation in larger-scale studies.

## 1. Introduction

Approximately 40% of virally suppressed people living with HIV infection (PLWH) show some degree of cognitive impairment, the etiology of which is not fully understood at present [1]. According to the Centers for Disease Control and Prevention, 15% of PLWH reported heavy drinking in the past month, which is a significant concern because alcohol has marked potential to exacerbate cognitive impairment in the context of HIV [2,3]. Emerging research suggests that pro-inflammatory perturbations of the gut-brain axis may contribute to neurocognitive dysfunction observed in HIV infection and alcohol use disorder [4,5]. The objective of the current study was to examine whether markers of gastrointestinal (GI) dysfunction and immune activation predict neurocognitive performance in heavy-drinking PLWH.

Independent of each other, HIV infection and heavy alcohol consumption have profoundly deleterious effects on the GI system. Both HIV and heavy drinking are known to deplete gut lymphocytes, disrupt tight junction proteins, and perturb the composition of the gut microbiome in favor of more pathogenic bacteria [6,7,8,9,10,11,12,13]. These mechanisms lead to microbial translocation, i.e., the abnormal movement of gut microbial products into systemic circulation, which can result in chronic immune activation and inflammation. A landmark study identified microbial translocation as a major contributor to chronic inflammation in PLWH [14]. Chronic inflammation persists even in PLWH who are virally suppressed on combination antiretroviral therapy (ART) and is associated with higher morbidity and mortality [15,16,17]. Moreover, a controlled alcohol administration experiment showed that a single binge-drinking episode caused microbial translocation and immune activation in healthy individuals [18]. In clinical settings, these phenomena are observed in individuals with chronic alcohol use disorder [19,20,21,22].

Microbial translocation can be assessed by measuring plasma levels of lipopolysaccharide (LPS, or endotoxin), a component of cell walls of Gram-negative bacteria [23]. LPS is a ligand for toll-like receptor 4 (TLR4), an innate immune receptor expressed on monocytes and macrophages [24]. Once LPS binds to the TLR4 receptor complex, activated monocytes initiate proinflammatory cytokine and chemokine production [25]. Markers of host response to microbial translocation include plasma levels of soluble cluster of differentiation 14 (sCD14), an acute phase protein shed from monocytes that facilitates binding of LPS to TLR4, and endotoxin core antibody immunoglobulin M (EndoCAb), an antibody in systemic circulation that binds to and clears LPS [24,26,27]. Elevated levels of sCD14 reflect monocyte activation in response to LPS and other ligands, whereas low levels of EndoCAb are interpreted as evidence of chronic immune stimulation by LPS [14,28].

Previously, our group reported that heavier alcohol use was associated with higher sCD14 levels in the current sample of 21 virally suppressed, heavy-drinking men living with HIV, independent of clinical and demographic factors [29]. Other groups have reported similar findings on alcohol and sCD14 [30,31]. However, in a larger study (of which the current sample represents a subset), severity of heavy drinking was not a strong predictor of neurocognitive deficits [32]. At the same time, several previous studies have linked microbial translocation and immune response to neurocognitive deficits and neural abnormalities in PLWH [33,34,35,36]. Therefore, the current analysis examined direct associations between inflammatory biomarkers (LPS, sCD14, EndoCAb) and neurocognitive performance. We hypothesized that higher LPS, higher sCD14, and lower EndoCAb would be associated with lower standardized scores on neurocognitive tests of learning, memory, processing speed, executive function, and verbal fluency. We found that higher LPS, higher sCD14, and lower EndoCab individually were associated with poorer processing speed, controlling for average weekly drinking and education. When considering all variables simultaneously as predictors of processing speed, higher LPS and sCD14 were retained as significant predictors of impaired functioning.

## 2. Materials and Methods

The current study utilized behavioral data and plasma samples from a randomized controlled trial (RCT) to reduce heavy drinking in HIV-infected men who have sex with men. The RCT was funded by the National Institutes on Alcohol Abuse and Alcoholism (NIAAA) and was conducted from 2010–2015 (P01AA019072; PI: C. Kahler). Inclusion criteria for the RCT were: (1) male; (2) 18 years of age or older; (3) had drank heavily in the past month, according to NIAAA guidelines (≥5 drinks per day, on one or more days; or >14 drinks per week, on average [37]); (4) confirmed diagnosis of HIV infection; and (5) self-reported sex with a male partner in the past 12 months. Exclusion criteria for the RCT were (1) current intravenous drug use; (2) current psychosis, suicidality, or mania, determined with the Structured Clinical Interview for DSM-IV-TR Axis I Disorders [38]; (3) treatment within past three months for an HIV-related opportunistic infection; (4) current psychotherapeutic or pharmacologic treatment for alcohol or drug use; and (5) score > 7 on Clinical Institute Withdrawal Assessment for Alcohol [39]. The secondary analysis reported here utilized blood samples and cognitive data collected at baseline and Month 3 assessment visits of the RCT.

Participants were asked to abstain from alcohol for 24 h before assessment, and zero breath alcohol content was confirmed upon arrival using a handheld digital breath analyzer. Urine samples were obtained to test for the presence of illicit drugs. Participants provided informed consent in a format approved by the Institutional Review Boards of Brown University (protocol #1008000242) and Fenway Health (protocol #347079-21).

For neurocognitive assessment, participants completed a brief cognitive battery to assess learning (List Recall of the Hopkins Verbal Learning Test-Revised [40]), memory (Delayed List Recall of the Hopkins Verbal Learning Test-Revised), processing speed (1—Trailmaking Test A [41]; 2—Digit Symbol Coding of the Wechsler Adult Intelligence Scale—third edition [42]), executive function (Trailmaking Test B [41]), and verbal fluency (Controlled Oral Word Association Test [43]). Standardized scores used in statistical analyses corrected for age and various other demographic factors such as race, depending on the norms published by the test creators. Years of education was included as a covariate in analyses because education was not an adjustment factor in all standardized scores.

The Timeline Followback Interview (TLFB) [44] was used to assess the number of standard alcoholic drinks (12 oz. of beer, 5 oz. of wine, 1.5 oz. 80-proof liquor) consumed each day in the 30 days preceding each assessment visit. Average drinks per week was calculated from the TLFB and was used as a covariate in statistical analyses. Our analysis on the association of alcohol use with immune biomarkers was reported previously [29].

For plasma assays, blood samples were processed upon collection, and plasma was aliquoted into endotoxin-free cryovials and stored at 80 °C until tested in batches. Enzyme-linked immunosorbent assays (ELISA) to quantify LPS (MyBiosource, San Diego, CA, USA), sCD14 (Enzo Life Sciences, Farmingdale, NY, USA), and EndoCAb (Hycult, Plymouth Meeting, PA, USA) were performed according to manufacturer instructions. Data were unavailable for three participants on LPS and for one participant on sCD14 and EndoCAb.

Generalized estimating equation (GEE) models [45] were used to test the time-varying associations of biomarker levels with cognitive performance. By accounting for correlations between repeated measures, GEE models allowed data from both time points (i.e., baseline and Month 3 of follow-up) to be analyzed simultaneously. First, individual models tested each biomarker as a predictor of standardized cognitive scores, with average drinks per week and education as covariates; Second, individual biomarker models were repeated, additionally controlling for (1) smoking status; (2) positive urine drug screen for marijuana (i.e., tetrahydrocannabinol); or (3) positive urine drug screen for other drugs (benzodiazepines, methamphetamine, opiates, cocaine, phencyclidine, barbiturates, methadone, ecstasy, oxycodone). For the “other drug” analysis, we created a composite to indicate positive results for one or more drug, due to low numbers testing positive for each individual drug type; Finally, because each of the three biomarkers individually predicted processing speed, we tested all biomarkers simultaneously as predictors of processing speed. For all analyses, alpha was set at *p* < 0.008 as Bonferroni adjustment for multiple comparisons. LPS was log-transformed to address high positive skew in its distribution.

## 3. Results

### 3.1. Participant Characteristics

Clinical and demographic characteristics of participants are shown in Table 1. All participants were heavy drinkers at baseline. Mean scores on cognitive tests were in the low-normal to normal range, yet there was considerable individual variability in scores. Percentages of positive urine screens for specific drugs were similar across baseline and follow-up and were as follows: marijuana, 24%; benzodiazepines, 12%; methamphetamine, 0%; opiates, 7%; cocaine, 17%; phencyclidine, 0%; barbiturates, 0%; methadone, 0%; ecstasy, 0%; oxycodone, 10%.

### 3.2. Predicting Cognitive Performance from Inflammatory Biomarkers

GEE models tested LPS, sCD14, and EndoCAb levels from plasma samples obtained at baseline and Month 3 as predictors of cognitive scores from the same time points.

#### 3.2.1. Individual Biomarkers

##### LPS

LPS was a significant predictor of the Digit Symbol test of processing speed, as was average drinks per week, but not education (Table 2, Model 1). Higher LPS levels were associated with lower Digit Symbol scores. LPS also predicted Trails B (Wald *χ*^2^(1) = 13.16, *p* = 0.0002, B = 12.18, 95% confidence interval (CI) (5.60, 18.76)), but in the opposite of the expected direction (i.e., higher LPS corresponded to better performance). LPS was not a significant predictor of other cognitive scores.

##### sCD14

sCD14 level was a significant predictor of the Digit Symbol test of processing speed, whereas average drinks per week and education were not significant predictors (Table 2, Model 2). Higher levels of sCD14 were associated with lower Digit Symbol scores. sCD14 was not a significant predictor of other cognitive scores.

##### EndoCAb

EndoCAb was a significant predictor of the Digit Symbol test of processing speed, while average drinks per week and education were not significant predictors (Table 2, Model 3). In addition, EndoCAb was a significant predictor of the Trails A test of processing speed (Wald *χ*^2^(1) = 7.41, *p* = 0.006, B = 0.21, 95% CI (0.06–0.35)). In both analyses, higher EndoCAb levels were associated with better processing speed scores, as predicted. EndoCAb did not significantly predict other cognitive scores.

#### 3.2.2. Individual Biomarkers, Controlling for Smoking Status, Marijuana Use, and Other Drug Use

##### Smoking Status

Because we previously found that smoking was negatively associated with processing speed in heavy-drinking men living with HIV [32], analyses were repeated with inclusion of smoking as a covariate. The significance of biomarker results was not altered by inclusion of smoking in the models.

##### Marijuana Use

In individual biomarker models controlling for marijuana use, the above results were unchanged, with the caveat that the positive association of EndoCAb with Trails A was reduced to trend level (*p* = 0.013).

##### Other Drug Use

In individual biomarker models controlling for other drug use, the above results were unchanged, except that the positive association of EndoCAb with Trails A became a marginal trend (*p* = 0.008).

#### 3.2.3. All Biomarkers

In the model with all biomarkers, average drinks per week and education as predictors of the Digit Symbol test of processing speed, higher levels of LPS and sCD14 remained significant predictors of worse performance (Table 3).

## 4. Discussion

In heavy-drinking men living with HIV, higher levels of microbial translocation and associated immune activation predicted worse cognitive processing speed. In data analyses, these associations were not explained by education, age, or the direct effect of alcohol use. Notably, all participants had been prescribed ART and had achieved viral suppression at the time of the study. Therefore, neither immune activation nor cognitive impairment appear to be due to direct effects of measurable viral replication as quantified by plasma HIV RNA assays. Rather, findings suggest that gut dysfunction may contribute to cognitive impairment in PLWH via systemic immune activation and inflammation. This study adds to growing evidence that the gut-brain axis plays a significant role in neurocognitive status in PLWH.

As brain cells that regulate neuroimmune response, particularly microglia, are sensitive to peripheral immune activation along the LPS pathway [46], the selected biomarkers (LPS, sCD14, EndoCAb) have clear relevance to brain health and functioning. However, it is not clear at present why these biomarkers were selectively associated with processing speed, but not with other neurocognitive domains. One could speculate that the selective association may reflect the type of brain tissue most affected by HIV- and alcohol-related inflammation. Neurocognitive processing speed is mediated predominantly by the health of the brain’s white matter [47,48]. Because both HIV and heavy drinking are associated with marked abnormalities in white matter with synergistic effects in co-occurring HIV and heavy drinking [49,50,51,52], it is possible that associations of immune biomarkers with processing speed in this sample are reflective of white matter damage related to chronic inflammation. Future studies examining associations of immune biomarkers with both cognition and white matter health would be informative.

All biomarkers predicted the Digit Symbol test of processing speed, whereas only EndoCAb predicted the Trails A test of processing speed. This discrepancy may be due to the fact that the Digit Symbol test is a more cognitively complex test of processing speed than Trails A, making it more feasible to detect associations with biomarkers. Although Trails B also is more complex than Trails A, Trails B is thought to tap into executive function rather than processing speed per se.

LPS was positively associated with executive function (Trails B), a finding that we recommend be interpreted with caution. This result may be a chance finding, despite statistical correction for multiple comparisons, or it might reflect the notion that LPS levels are most informative when interpreted in the context of host response. Experimental and clinical studies indicate that LPS is a useful marker of microbial translocation in the context of HIV infection [14,53]. However, sCD14 and other markers of host response tend to be stronger predictors of cognitive impairment and mortality in PLWH [17,54]. An analysis of plasma and cerebrospinal fluid (CSF) from 62 PLWH found that LPS was not present in CSF samples, whereas sCD14 was detected in all CSF samples [55]. At the same time, plasma LPS was significantly correlated with sCD14 in CSF, underscoring the ultimate importance of the LPS pathway [55]. Taken together, these findings suggest that microbial translocation is a critical phenomenon to assess but that host response to microbial translocation may be a more reliable predictor of key clinical outcomes.

This study is limited by relatively small sample size, lack of a control group, and the all-male sample. It was designed as a pilot study to gather preliminary data on associations among alcohol use, inflammation, and cognition in PLWH, hence the absence of a control group. Results should be considered preliminary pending replication. Because chronic inflammation is a core feature of HIV infection, an important unanswered question is whether the observed associations between inflammatory biomarkers and processing speed are specific to PLWH who drink heavily or are present in the general population of PLWH. Much previous research on inflammation and cognition in PLWH has examined associations with global cognitive performance, rather than specific domains, e.g., [33,35,56]. Two studies reported associations of sCD14 with various other cognitive domains (verbal learning, executive functioning, psychomotor speed, attention, or learning) in PLWH, but samples differed widely in substance use characteristics [36,54]. In future full-scale studies, it is recommended to compare specific cognitive domains in demographically similar seropositive and seronegative individuals with a full range of drinking behavior (i.e., nondrinkers, moderate drinkers, heavy drinkers), in order to parse the individual contributions of HIV status and alcohol use. Additionally, longitudinal studies are warranted to assess whether changes in alcohol intake (e.g., decreasing heavy drinking) may ameliorate the chronic inflammatory processes documented in the current study.

In our previous study on this virally suppressed sample of heavy-drinking men living with HIV, we reported that alcohol quantity and frequency, but not HIV clinical variables, were associated with sCD14, a marker of monocyte activation [29]. The current study found that sCD14, as well as LPS and EndoCAb, predicted cognitive processing speed performance. Several other studies have reported that sCD14 independently predicts mortality as well as cognitive impairment in PLWH [17,54,57,58]. Together, these findings suggest that minimizing alcohol use may be critical for reducing risk of inflammation-related cognitive impairment and mortality in PLWH. In conclusion, the gut-brain axis warrants exploration in future studies to advance understanding of and treatment for neurocognitive dysfunction in HIV infection.

## Figures and Tables

**Table 1:** Baseline participant demographic and clinical characteristics (*N* = 21).

	Mean (±Standard Deviation) or Percent	Range
Age (years)	46.7 ± 8.5	26–63
Education (years)	15.1 ± 2.9	12–22
Duration of HIV (years)	12.2 ± 9.2	0.4–28.2
Current smokers	66%	---
On ART	100%	---
Viral suppression ≤75 copies/mL	100%	---
CD4 T-cell count	643 ± 245	222–1156
Average drinks/week at baseline	22.1 ± 16.0	7.5–84.4
Learning ^1^—List Recall	39.1 ± 10.3	20–63
Memory—Delayed List Recall	43.9 ± 9.7	25–61
Processing Speed—Trails A	50.7 ± 7.9	34–61
Processing Speed—Digit Symbol	43.4 ± 8.5	33–63
Executive Function—Trails B	40.8 ± 18.5	9–70
Verbal Fluency—Controlled Oral Word Assoc.	49.1 ± 8.7	31–64

^1^ Standardized neurocognitive scores are expressed as *t*-scores with mean = 50 and standard deviation = 10.

**Table 2:** Results of individual GEE models predicting processing speed (WAIS Digit Symbol test) using immune biomarkers, average weekly drinks, and education.

**Model 1: LPS**	**B ^1^**	**95% CI**	**Wald *χ*^2^**	***p*-Value**
LPS **	−7.02	−10.85, −3.19	12.89	0.0003
Average drinks/week **	−0.27	−0.42, −0.12	12.61	0.0004
Education	0.82	−0.87, 2.51	0.91	0.341
**Model 2: sCD14**	**B**	**95% CI**	**Wald *χ*^2^**	***p*-Value**
sCD14 **	−0.003	−0.004, −0.002	20.69	<0.0001
Average drinks/week	0.06	−0.01, 0.13	3.17	0.075
Education	0.37	−0.84, 1.58	0.36	0.547
**Model 3: EndoCAb**	**B**	**95% CI**	**Wald *χ*^2^**	***p*-Value**
EndoCAb **	0.25	0.13, 0.37	16.70	<0.0001
Average drinks/week	−0.05	−0.10, 0.01	2.58	0.108
Education	1.31	0.24, 2.38	5.72	0.017

^1^ In all models, beta coefficients represent change in the outcome for a 1-unit change in the predictor. ** Indicates significant predictor at *p* < 0.008.

**Table 3:** Results of GEE model predicting processing speed (WAIS Digit Symbol test) using all immune biomarkers, average weekly drinks, and education

Predictor	B	95% CI	Wald *χ*^2^	*p*-Value
LPS **	−6.23	−8.73, −3.73	23.86	<0.0001
sCD14 **	−0.003	−0.004, −0.002	29.91	<0.0001
EndoCAb	0.04	−0.20, 0.28	0.09	0.768
Average drinks/week	−0.09	−0.22, 0.04	1.71	0.192
Education	0.23	−1.36, 1.82	0.08	0.773

** Indicates significant predictor at *p* < 0.008.
